# Enhancement of *Campylobacter hepaticus* culturing to facilitate downstream applications

**DOI:** 10.1038/s41598-021-00277-8

**Published:** 2021-10-21

**Authors:** Canh Phung, Timothy B. Wilson, José A. Quinteros, Peter C. Scott, Robert J. Moore, Thi Thu Hao Van

**Affiliations:** 1 School of Science, RMIT University, Bundoora West Campus, Bundoora, VIC Australia; 2 Scolexia Pty. Ltd., Moonee Ponds, VIC Australia

**Keywords:** Biological techniques, Biotechnology, Computational biology and bioinformatics, Microbiology

## Abstract

*Campylobacter hepaticus* causes Spotty Liver Disease (SLD) in chickens. *C. hepaticus* is fastidious and slow-growing, presenting difficulties when growing this bacterium for the preparation of bacterin vaccines and experimental disease challenge trials. This study applied genomic analysis and in vitro experiments to develop an enhanced *C. hepaticus* liquid culture method. In silico analysis of the anabolic pathways encoded by *C. hepaticus* revealed that the bacterium is unable to biosynthesise l-cysteine, l-lysine and l-arginine. It was found that l-cysteine added to Brucella broth, significantly enhanced the growth of *C. hepaticus*, but l-lysine or l-arginine addition did not enhance growth. Brucella broth supplemented with l-cysteine (0.4 mM), l-glutamine (4 mM), and sodium pyruvate (10 mM) gave high-density growth of *C. hepaticus* and resulted in an almost tenfold increase in culture density compared to the growth in Brucella broth alone (log10 = 9.3 vs 8.4 CFU/mL). The type of culture flask used also significantly affected *C. hepaticus* culture density. An SLD challenge trial demonstrated that *C. hepaticus* grown in the enhanced culture conditions retained full virulence. The enhanced liquid culture method developed in this study enables the efficient production of bacterial biomass and therefore facilitates further studies of SLD biology and vaccine development.

## Introduction

*Campylobacter hepaticus* has been identified as the causative agent of Spotty Liver Disease (SLD) in laying hens^1^. *C. hepaticus* is a fastidious bacterium that requires microaerobic conditions (a mix of 5–10% oxygen, 5–10% CO_2,_ and 80–85% N_2_), a narrow temperature range (growth at 37 and 42 °C, but not 25 °C), and rich nutrient media for growth^1–4^. The incubation time needed to form *C. hepaticus* colonies on agar plates varies from 3 to 7 days, depending on the strain cultivated^1,2,5^. The incubation time for other *Campylobacter* species, such as *C. jejuni, C. coli, C. lari*, and *C. concisus* ranges from 24 to 48 h on plates and 18–24 h in liquid media^6–9^. In previous studies*,* when a large *C. hepaticus* biomass was required, such as for SLD induction experiments, *C. hepaticus* was cultured on Brucella agar supplemented with 5% defibrinated horse blood (HBA), incubated for 3 days and harvested. *C. hepaticus* had to be harvested from dozens of Petri dishes to produce sufficient biomass for a modestly sized animal trial^10^. This methodology is time–consuming, uses a lot of resources, and is prone to contamination. It also presents difficulties with scaling up to produce sufficient biomass to produce challenge material for large animal trials or for production of killed vaccines*.*

Although Brucella broth, the standard media for growing *C. hepaticus* cultures, is a rich medium, it may not supply all the nutrients required to support optimal growth of *C. hepaticus*. An in silico approach was used to identify nutrients that may need to be supplemented to improve culture productivity. The availability of *C. hepaticus* whole-genome sequences^11^ and tools such as Metagenomic Rapid Annotations using Subsystem Technology (MG-RAST)^12^ and Kyoto Encyclopedia of Genes and Genomes (KEGG)^13^ to annotate genomes and analyse metabolic pathways, allows the identification of growth-supporting compounds for *C. hepaticus*. KEGG analysis has been used to predict the nutritional requirements of *C. jejuni* NCTC 11168^14^. The study found that l-cysteine, l-leucine, l-methionine, and l-aspartic acid are essential amino acids that need to be exogenously supplied for the growth of *C. jejuni*. The addition of pyruvate or lactate and niacinamide as carbon sources have previously been shown to improve the growth of *C. jejuni* NCTC 11168^15^. Similarly, necessary substrates for the growth of *Bukholderia glumae* were defined using the Pathcomp tool in KEGG^16^*.*

The objective of this project was to identify and evaluate compounds that are required or that could enhance the growth of *C. hepaticus* in liquid culture*,* by analysing the metabolic pathways of this species. Also, culture conditions, including temperature, pH, mixing, and culture vessel types were assessed to characterise and improve the growth of *C hepaticus* in liquid culture. A reliable liquid culture method that resulted in high culture biomass would aid in the design of reproducible assays to investigate stress resistance, virulence mechanisms, vaccine development, and survival in the environment of *C. hepaticus*. This study applied genomic analysis and in vitro experiments to develop an enhanced *C. hepaticus* liquid culture method.

## Results

### In silico pathway analysis of *C. hepaticus*

**Figure 1:**
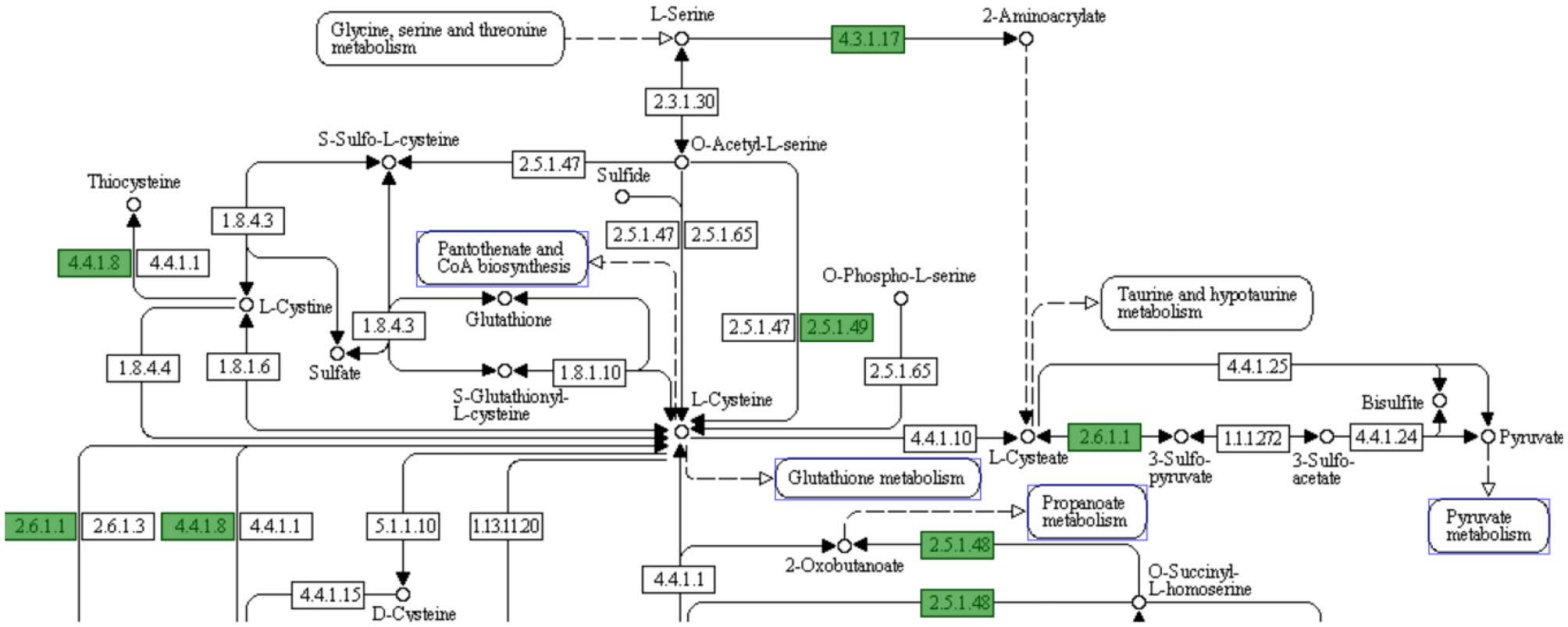
KEGG pathway of l-cysteine of *C. hepaticus*. Green boxes indicate the genes identified in *C. hepaticus* while white boxes show absent genes. *C. hepaticus* lacks genes including *cysE* (EC 2.3.1.30, serine acetyltransferase), *cysK* (EC 2.5.1.47, cysteine synthase A), *cysM* (EC 2.5.1.65, *O*-phosphoserine sulfhydrylase) and *cys3* (EC 4.4.1.1, cystathionine γ-lyase).

KEGG pathway analysis of three *C. hepaticus* strains showed that this bacterium harbours a complete tricarboxylic acid (TCA) cycle. Therefore, *C. hepaticus* can use all substrates in the citric pathways including pyruvate, succinate, oxaloacetate, fumarate, 2-oxoglutarate, malate and citrate as energy sources. The genome of *C. hepaticus* also contains genes encoding the enzymes for complete metabolic pathways for many amino acids, such as l-methionine, l-histidine, l-alanine, l-glycine, l-valine, l-leucine and l-threonine. In contrast, *C. hepaticus* lacks a complete pathway for the biosynthesis of l-arginine from l-glutamate. The *argE* gene, encoding acetylcitrulline deacetylase is absent. l-lysine cannot be biosynthesised from l-aspartate as the *dapA* and *dapB* genes are not present. Similarly, it is predicted that l-cysteine cannot be biosynthesised, as the *cysE, cysM* and *cysK* genes required to synthesise l-cysteine from l-serine and l-methionine, are absent (Fig. 1). Consequently, *C. hepaticus* was predicted to be unable to biosynthesise l-cysteine, l-lysine and l-arginine. Adding these amino acids to the culture media may improve the growth of *C. hepaticus.* This prediction was tested in the following in vitro experiments. Sodium pyruvate was also added into Brucella broth to check the growth of *C. hepaticus,* because it has been used as a carbon source^14^ and scavenges hydrogen peroxide^17^, an oxidative stress factor that is generated via the use of oxygen of bacteria^18^.

### The effect of culture vessel type, pH, and inoculum level on the yield of *C. hepaticus*

**Figure 2:**
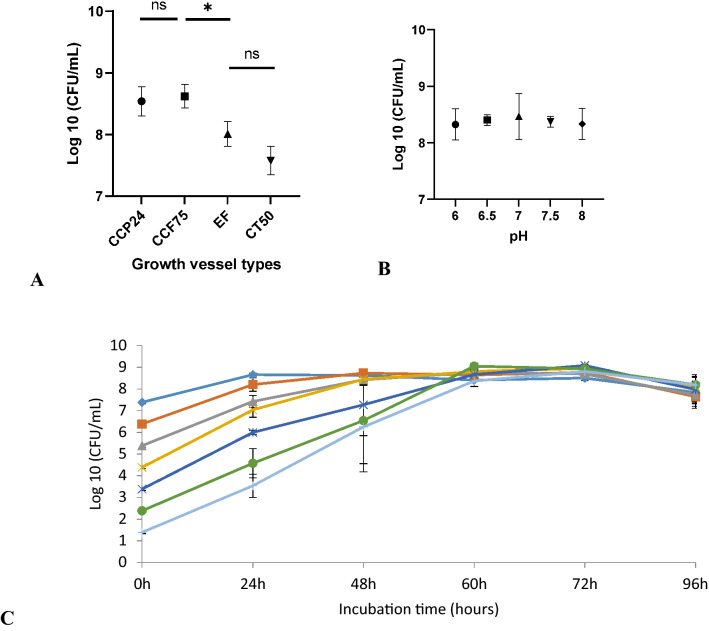
**(A)** Effect of growth vessel type on the growth of *C. hepaticus* HV10^T^. *CCP24* 24 well cell culture flask, *TCF75* tissue culture flask 75cm^2^, *EF* Erlenmeyer flask, *CT50* corning 50 mL tube. **(B)** Growth of *C. hepaticus* in Brucella broth pH adjusted 6–8 after 48 h of incubation. **(C)** Kinetic growth of *C. hepaticus* in Brucella broth. The initial inoculum was from 10^1^ to 10^7^ CFU/mL. Viable cells were counted after 0, 24, 48, 60, 72 and 96 h of incubation. P values were calculated using unpaired t-tests. Significant difference: * p ≤ 0.05, *ns* no significance. Data points represent results of 4 biological replicates.

Before testing the effect of amino acids and sodium pyruvate on the growth of *C. hepaticus*, three factors, culture vessel types, pH, and inoculum level were examined. Figure 2A shows that *C. hepaticus* grew to a higher final density when growth in 24-well cell culture plates (CCP24) and 75 cm^2^ cell culture flasks (TCF75) (log10 CFU/mL = 8.54–8.62 (P ≤ 0.05) compared to that seen in 50 mL tubes (CT50) and 250 mL Erlenmeyer flasks (EF) (log CFU/mL = 7.75–8.0). The CCP24 culture plates (1 mL of culture added/well) were therefore used to test the effect of amino acid and sodium pyruvate supplementation on the growth of *C. hepaticus,* and TCF75 flasks (25 mL of media/flask) were used to scale-up the production of biomass of *C. hepaticus.*

*C. hepaticus* was monitored for growth in Brucella broth adjusted to a pH ranging from 6.0 to 8.0 to evaluate the pH tolerance. There was no statistically significant difference in the growth of *C. hepaticus*, indicating little or no effect of pH within this range (Fig. 2B). Therefore, Brucella broth without pH adjustment was used for the further culturing of *C. hepaticus,* as it has a neutral pH value (7.0 ± 0.2)*.*

The growth kinetics of *C. hepaticus* in Brucella broth was investigated using initial inocula ranging from 10^1^ to 10^7^ CFU/mL (Fig. 2C). Viable cells counts were carried out after 0, 24, 48, 60, 72 and 96 h of incubation. The two highest inoculum levels (10^6^ and 10^7^ CFU/ml) produced maximum growth to 10^8^ CFU/ml after 24 h of incubation. With the lower inoculum levels (10^5^ and 10^4^ CFU/mL), *C. hepaticus* achieved maximum growth after 48 h of incubation and for low inoculum levels of 10^1^ to 10^3^ CFU/ml, the maximum growth of *C. hepaticus* was reached after 60 h.

### The effect of amino acids and sodium pyruvate on the growth of *C. hepaticus*

**Figure 3:**
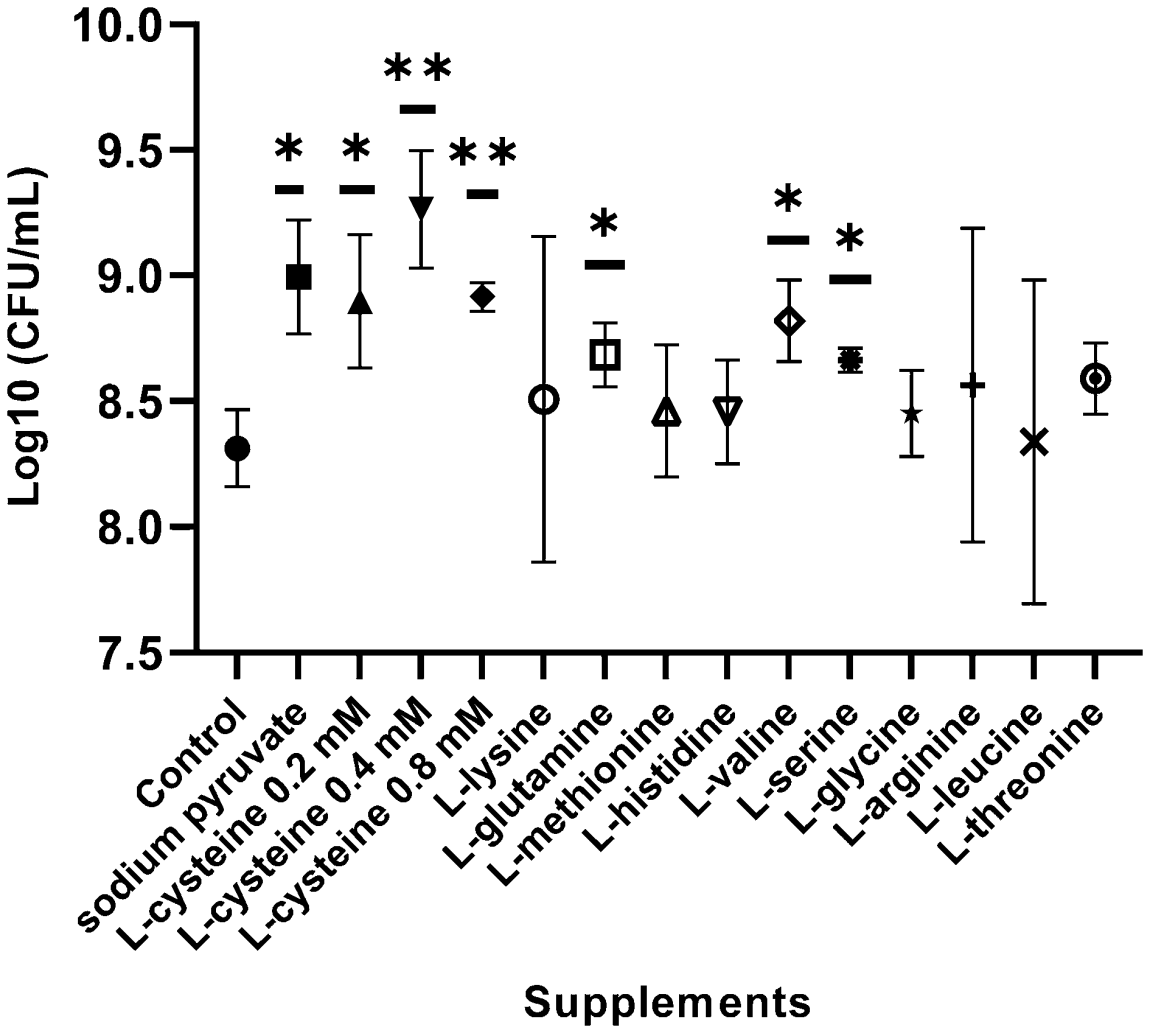
Effect of sodium pyruvate and amino acids on the growth of *C. hepaticus* HV10^T^ in Brucella broth (control). Cultures were inoculated with 10^6^ CFU/ml and incubated for 48 h at 37 °C. P-values were calculated using unpaired t-tests. Significant difference *: p < 0.05; **: p < 0.005*.*

*C. hepaticus* cultured in Brucella broth supplemented with 0.4 mM l-cysteine, 4 mM l- glutamine, 0.8 mM l-valine, 0.4 mM l-serine and 10 mM sodium pyruvate exhibited a significantly greater growth compared to a culture grown in Brucella broth only (log10 = 8.66–9.26 vs 8.31 CFU/mL) (Fig. 3). l-cysteine (0.4 mM) showed highest growth enhancement to log10 = 9.26 CFU/mL, significantly higher than the density obtained with the other supplements (p ≤ 0.005). No significant difference was observed (p > 0.05, Fig. 3) when *C. hepaticus* was grown in Brucella broth supplemented with other amino acids including l-lysine, l-methionine, l-histidine, l-glycine, l- arginine, l-leucine, and l-threonine compared to a culture grown in Brucella broth only.

The growth of *C. hepaticus* was then investigated in Brucella broth supplemented with different combinations of each compound that could enhance the growth of *C. hepaticus,* as demonstrated above*,* including l-cysteine, l-glutamine, l-valine, l-serine, and sodium pyruvate. In general, all combinations of supplements in Brucella broth significantly enhanced the growth of *C. hepaticus* compared to Brucella broth alone. The highest growth of *C. hepaticus* observed was in Brucella broth supplemented with a mixture of l-cysteine (0.4 mM), l-glutamine (4 mM) and sodium pyruvate (10 mM) (log10 = 9.34), followed by a combination of l-cysteine (0.4 mM) and sodium pyruvate (10 mM) (log10 = 9.17), and only l-cysteine (0.4 mM) (log10 = 9.11) (Table 1). Brucella broth supplemented with l-cysteine (0.4 mM), and l-glutamine (4 mM) and sodium pyruvate (10 mM) was then used to grow *C. hepaticus* in TCF75 flasks in the following experiments.

**Table 1:** The effects of different supplements added to Brucella broth on the growth of *C. hepaticus* compared to Brucella broth only.

Medium	Log10 (CFU/mL)	SEM	P value	Significant difference
Brucella broth (reference)	8.42	0.10		
Brucella broth + l-cysteine	9.11	0.05	0.0008***	Yes
Brucella broth + l-cysteine + sodium pyruvate	9.17	0.07	0.0009***	Yes
Brucella broth + l-cysteine + l-glutamine	9.04	0.10	0.0049**	Yes
Brucella broth + l-cysteine + l-glutamine + sodium pyruvate	9.34	0.08	0.0003***	Yes
Brucella broth + l-cysteine + Sodium pyruvate + l-valine	9.03	0.03	0.0012**	Yes
Brucella broth + l-cysteine + Sodium Pyruate + l-serine	9.01	0.18	0.0278*	Yes

Cultures were inoculated with 10^6^ CFU/ml and incubated for 48 h at 37 °C. One way ANOVA followed by Dunnett’s multiple comparison test showed that the differences between all the additive groups and the reference group (unsupplemented Brucella broth) were all highly significantly different. The P-values presented are more conservative values calculated using unpaired t-tests.

*SEM* standard error of mean, *CFU* colony-forming unit; significant difference.

*: p ≤ 0.05; **: p ≤ 0.01; ***: p ≤ 0.001.

### Effect of temperature and incubating conditions on growth of *C. hepaticus*

Because the culture vessels that provided the largest surface to volume ratio produced the highest density cultures it was proposed that gas exchange between the liquid medium and the microaerophilic atmosphere might be important. Therefore, the effects of agitation of the cultures by shaking were investigated. In the modified Brucella broth developed in this study, after 48 h of incubation *C. hepaticus* grew significantly better (p ≤ 0.05) in static conditions, with log10 = 9.25 CFU/mL compared to log10 = 8.76 CFU/mL when shaken. It was also found that *C. hepaticus* exhibited significantly better growth at 37 °C than 42 °C (p ≤ 0.05), log10 = 9.28 CFU/mL compared to log10 = 8.09 CFU/mL.

### Growth of different *C. hepaticus* strains in modified Brucella broth

**Figure 4:**
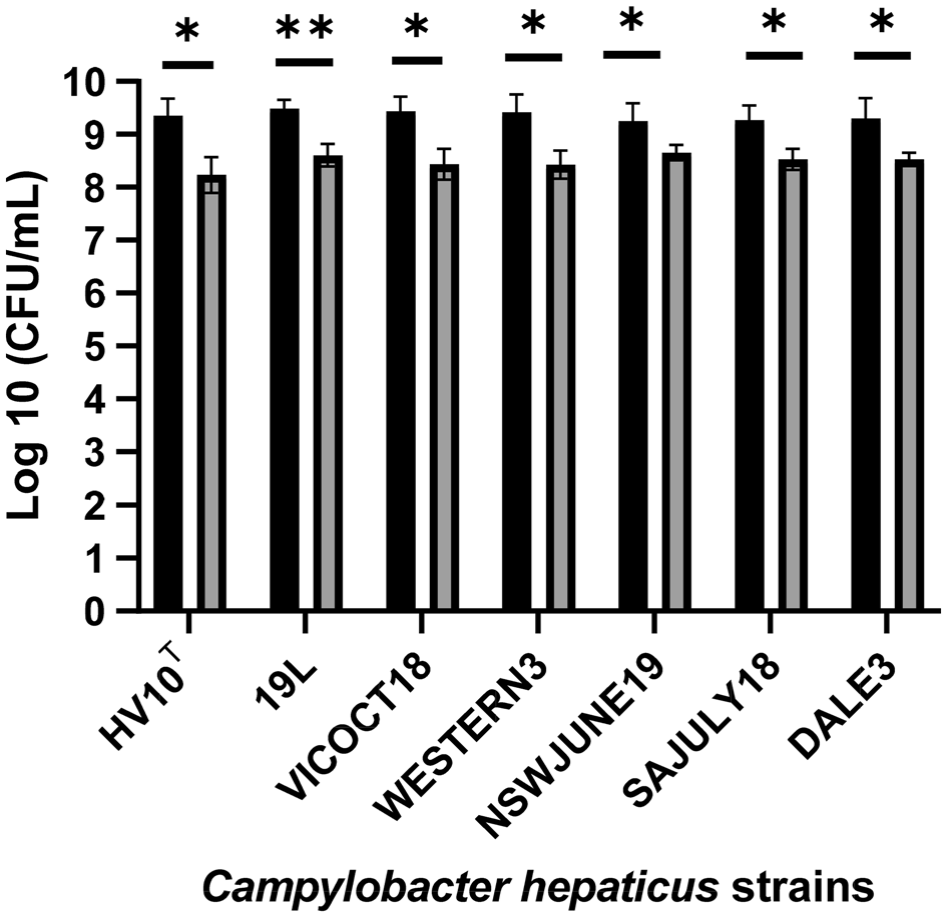
Growth of *C. hepaticus* isolates in modified Brucella broth (Brucella broth supplemented with 0.4 mM l-cysteine, 4 mM l-glutamine and 10 mM sodium pyruvate) compared to non-supplemented Brucella broth after 48 h of incubation under microaerobic conditions. Solid black bars illustrate the growth of *C. hepaticus* strains in modified Brucella broth, grey bars in Brucella broth only. P values were calculated using unpaired t-tests. Significant difference *: p ≤ 0.05. **: p ≤ 0.01.

The medium development experiments described above were carried out on a single strain of *C. hepaticus*. To determine if the improved media composition could also enhance the growth of other *C. hepaticus* isolates, the growth of different isolates in the modified Brucella broth**,** at 37 °C, under microaerobic conditions, in static TCF75 flasks was examined**.** All tested strains of *C. hepaticus* (HV10^T^, 19L. VICOCT18, WESTERN3, NSWJUNE19, SAJULY18 and DALE3) reached densities of log10 = 9.25–9.49 CFU/mL after 48 h, significantly higher (p ≤ 0.05) than non-supplemented Brucella broth (log10 = 8.24–8.66 CFU/mL) Fig. 4). No significant difference in growth was observed among all *C. hepaticus* isolates in the modified Brucella broth.

### Experimental infection of laying hens with *C. hepaticus* culture grown in modified Brucella broth

**Figure 5:**
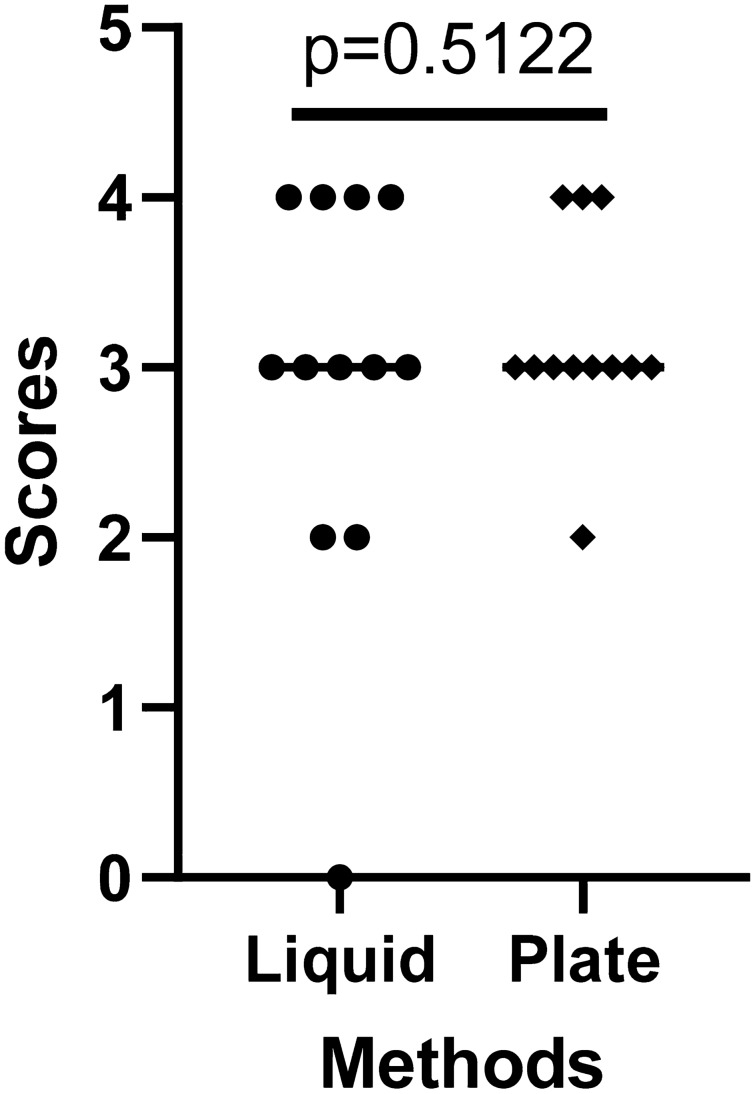
SLD spot scores of SLD-positive laying hens after challenge with *C. hepaticus* HV10^T^ grown from modified Brucella broth (liquid method) and HBA plate method based on scores of spots. This score is based on a logarithmic scale from 0 to 4, where 0 = no visible lesions, 1 = 1–9 lesions, 2 = 10–99 lesions, 3 = 100–999 lesions and 4 = more than 1000 lesions. P values were calculated using unpaired t-tests.

One of the principal reasons to develop improved culturing conditions for *C. hepaticus* was to make the preparation of challenge inocula easier and more reproducible for experimental infection trials used to investigate the biology of infections and assess the efficacy of various SLD treatment protocols. Experience with preparing challenge inocula using the previously described plate harvesting method had suggested that the state of the inoculum was critical to the success of infection^10^. Therefore, it was important to establish that the cultures grown in the modified Brucella liquid medium were capable of eliciting disease. Hens entering peak lay were challenged with, either *C. hepaticus* HV10^T^ harvested from Brucella agar plates, or bacteria grown in the newly devised liquid media conditions and the induction of SLD lesions on the liver were scored. Birds in the control group, inoculated with fresh modified Brucella broth, showed no lesion on livers, whereas 12 out of 12 hens inoculated with the agar plate derived bacteria had lesions on the liver and 11 out of 12 hens inoculated with bacteria grown in the modified Brucella liquid had liver lesions. Based on disease scores, there was no significant difference between the degree of disease elicited by bacteria grown under the two conditions (Fig. 5), demonstrating that *C. hepaticus* grown in the modified Brucella broth developed in this study had equal levels of virulence as those grown by the previously described plate culture method. The group sizes were sufficient to detect a one-point difference in mean scores with an alpha of 0.05 and 80% power.

## Discussion

In silico pathway analysis of the *C. hepaticus* genome predicted that the bacterium lacks some of the genes encoding enzymes required for the biosynthesis of l-cysteine, l-lysine and l-arginine. *C. hepaticus* lacks the genes *cysE* and *cysK, cysM* to synthesise l-cysteine from l-serine and sulphur. *cysE* encodes serine acetyltransferase and *cysK* encodes cysteine synthase. These enzymes are required for the synthesis of l-cysteine from l-serine in bacteria and plants^19^. CysE synthesises l-cysteine from l-serine by catalysing an acyl transfer from acetyl-CoA. CysK catalyses O-acetyl-l-serine combining with hydrogen sulphide to yield l-cysteine^19^. The addition of l-cysteine to Brucella broth improved the growth of *C. hepaticus* significantly, to more than 10^9^ CFU/mL compared to Brucella broth only. This indicates that even though Brucella broth is a rich nutrient source the l-cysteine level is insufficient to support maximal growth of *C. hepaticus*. Growth support of l-cysteine was also reported in other *Campylobacter* species. l-cysteine was defined as a vital source of sulphur for *C. jejuni*^20^. *C. jejuni* grown in minimal medium with l-cysteine added was better than their growth in the medium without cysteine^14,21^. l-cysteine has been identified as a chemotactic attractants of *C. jejuni*^22,23^. In contrast, although *C. hepaticus* was also predicted to be unable to biosynthesise l-lysine and l-arginine, the addition of these amino acids to Brucella broth did not significantly increase the culture densities that could be achieved for *C. hepaticus* compared to densities supported by unmodified Brucella broth. Similarly, the addition of l-arginine and l-lysine to minimal media did not enhance the growth of *C. jejuni* because *C. jejuni* may have genes that support the biosynthesis of l-arginine and l-lysine^14^. Better growth of *C. hepaticus* in Brucella broth supplemented with l-glutamine, l-valine or l-serine was observed although no missing genes that are responsible for the biosynthesis of these amino acids by *C. hepaticus* were detected. It was demonstrated that l-glutamine and l-serine are chemoattractants for *C. jejuni*^24^. In chickens, l-cysteine, l-glutamine, and l-serine are abundant in chicken liver. The chicken gut also has sufficent quantities of necessary amino acids such as l-cysteine, l-glutamine, l-valine and l-serine for the growth of many bacteria^25^ and this likely explains why, despite its inability to synthesise a number of amino acids, *C. hepaticus* can colonise the intestinal tract of laying hens^10^. It was found that the reduction of amino acids such as l-cysteine, l-glutamine and l-serine in the chicken diet contributed to the reduction of *Campylobacters* in chickens^26^. The authors explained that these amino acid are involved in the formation of the mucin production of the intestinal mucus layer and essential for the survival and growth of *Campylobacter*s^27^. Thus, an increase or decrease in the concentration of these amino acids results in changes in the number of *Campylobacters* in the chicken gut^26^. For other amino acids including l-methionine, l-histidine, l-glycine, l-leucine, and l-threonine, there was no significant difference in the growth of *C. hepaticus.* These results agree with the bioinformatic pathway analysis.

This study found that the *C. hepaticus* showed maximum growth in Brucella broth supplemented with a mixture of l-cysteine (0.4 mM), l-glutamine (4 mM) and sodium pyruvate (10 mM) and reached 10^9.34^ CFU/mL. Sodium pyruvate provides an additional carbon source to support the growth of *C. hepaticus* in Brucella broth. *C. hepaticus* may heavily depend on the Kreb's cycle to generate energy. Many studies have demonstrated the important role of pyruvate in the growth of campylobacters. Sodium pyruvate is one of the compounds in the *Campylobacter* growth supplement product (FBP supplement)^28^ and *Campylobacter* selective supplement^29^. Pyruvate was found to promote the growth of *C. jejuni* NCTC 11168 in MEM medium^14^. This compound plays a central role in *C. jejuni* metabolism and can be fermented to various products such as acetate, formate, lactate and succinate. It is linked to carbohydrate and amino acid catabolism to produce energy. The synthesis of l-leucine, l-valine, l-alanine and l-isoleucine by campylobacters use pathways in which pyruvate can play a key role^30^. Pyruvate also acts as an electron acceptor and can decrease the concentration of hydrogen peroxide to reduce the damage caused by oxygen to bacteria^31^.

*C. hepaticus* grew to higher densities in CCP24 and TCF75 than CT50 and EF, suggesting that the surface-area-to-volume ratio (S:V) may be important, possibly for gas exchange, as mentioned in a study of improvement of culturing of *C. jejuni*^32^. These authors demonstrated that TCF75 has more S:V ratio than EF and CT50 and therefore TCF75 provided better atmospheric exchange than EF and CT50. Also, tissue culture flasks were recommended to grow and study standard growth curves of *C. jejuni*^33^. In a study of the growth of *Helicobacter pylori*, Gas–Permeable Lifecell tissue culture flasks gave improved growth in Brucella broth supplemented with fetal bovine serum^34^. These authors also mentioned the effect of surface area on the growth of *H. pylori* and suggested that a small surface area resulted in poorer growth of *H. pylori*. Tissue culture flasks were used early in the history of the culture of *C. jejuni* and *C. pylori*^35,36^ and have been employed in many studies of *Campylobacter* species. It may show that *C. hepaticus* requires a larger S:V ratio to grow due to better gas exchange, although it is then unexpected that agitation had a negative effect. A low level of oxygen may be an obligatory requirement for *C. hepaticus* growth. Under anaerobic conditions and higher oxygen tensions (21%), *C. hepaticus* failed to grow^1^. Similarly, *C. jejuni* does not grow under anaerobic conditions^37,38^ and oxygen is considered a requirement for DNA synthesis in *C. jejuni*^37^. *Campylobacter* spp. are sensitive to high oxygen tensions, but still need an optimal oxygen concentration (2%-10%) to grow^38^. *C. hepaticus* contains genes for oxidative phosphorylation^11^ that need oxygen as an acceptor to generate ATP.

*C. hepaticus* achieved maximum growth after 48–72 h, depending on the initial inoculum, showing slower growth in comparison to other *Campylobacters*. The maximum growth of *C. jejuni* was at around 30 h in both Nutrient Broth Number 2 and Mueller Hinton broth^39^. *C. jejuni* reached the highest densities after 24 h of growth in Brain Heart Infusion broth^40^. *C. hepaticus* can grow in a range of pH from 6.0 to 8.0. It has been reported that other *Campylobacter* species also grow well in this pH range^41^. Chickens normally have a pH of around 6.3 in the liver^42^, 6.4 in the small and large intestine^43^, 6.6—6.7 for caecum^43,44^, and 6.0 for bile^45^. These are all tissues and environments in which it has been shown that *C. hepaticus* can survive and colonise. *C. hepaticus* showed better growth at 37 °C than 42 °C while this microorganism has only been isolated from chickens, which have a body temperature of 40–42°^o^C^46^. The growth of *C. hepaticus* was reported to be somewhat slow at 42 °C, taking 7 days to form colonies in sheep blood agar^3^. Temperature differently affects *Campylobacter* species regarding the growth, motility, and ability to invade the host cells. A study showed that *C. jejuni* grew at both 37 °C and 42 °C but showed differences in motility and invasion. *C. coli* grew and moved better at 42 °C. *C. fetus,* a bacterium that is frequently detected in poultry, showed greater growth and invasion at 37 °C^47^.

Using the modified Brucella broth developed in this study, together with growth conditions including the use of a large surface area culture vessel, at 37 °C, in microaerophilic and static conditions, *C. hepaticus* cultures could grow to 10^9^ CFU/mL and showed virulence in laying hens. This culturing method is time-saving and more cost-effective than the previously used plate harvesting method to obtain the large biomass required for SLD animal induction experiments and bacterin vaccine production. It also reduces the amount of subculturing needed, possibly minimising the effect of repeated subculture on the virulence of *C. hepaticus.* A study has shown that the repeated subculturing of the somewhat related organism, *H. pylori,* could result in a decrease of adhesion, motility, gastric inflammation and cytotoxicity, and repeated culturing is a recognised way that bacteria have been attenuated to produce live vaccines^48^. Thus, the method described in this study can facilitate further studies on *C. hepaticus* biology and SLD.

## Materials and methods

### In silico analysis of *C. hepaticus* metabolism

The metabolic pathways encoded within the genomes of *C. hepaticus* strains HV10^T^, 19L and VICOCT18, were analysed using (1) RAST (Rapid Annotation using Subsystem Technology)^12^ and the SEED^49^ (http://rast.nmpdr.org/rast.cgi) for annotating the genomes and metabolic pathways prediction of *C. hepaticus*; and (2) BLAST^50^ (Basic Local Alignment Search Tool) (https://blast.ncbi.nlm.nih.gov/Blast.cgi) for comparing nucleotides and protein sequences of *C. hepaticus* with available sequences in the gene bank. *C. hepaticus* HV10^T^ is the type strain for *C. hepaticus*, 19L is a representative of a clade that is distinct form HV10^T^ and VICOCT18 is a more recent isolate.

### Bacterial strains and culture conditions

The *C. hepaticus* strains HV10^T^, 19L^11^, VICOCT18, WESTERN3, NSWJUNE19, SAJULY18 and DALE3^51^ were used in the study. These strains are representative isolates from independent SLD outbreaks from widely separated geographical locations in Australia. All *C. hepaticus* strains were stored at − 80 °C in 70% Brucella broth (BD BBL™) and 30% glycerol. *C. hepaticus* was routinely cultured on Brucella agar plates (Brucella broth (BD BBL™) + 1.5% agar (BD BBL™)) supplemented with 5% defibrinated horse blood (Equicel) (HBA) and cultured at 37 °C under microaerobic conditions (created using Campygen 3.5L gas generation packs (Oxoid)) in an anaerobic jar, for 96 h to recover *C. hepaticus* cells from − 80 °C stock or for 72 h if they were subcultured from HBA plates.

### Effect of type of culture vessel on the growth of *C. hepaticus*

Costar^®^ 24-well cell culture plates (CCP24), Corning^®^ 50 mL centrifuge tubes (CT50) with a vented cap (0.2 μm pore size), Corning^®^ 75cm^2^ cell culture flask (TCF75) with a vented cap (0.2 μm pore size) and Erlenmeyer flasks (250 mL) (EF) were used to compare the growth of *C. hepaticus* in Brucella broth (BD BBL™). The volume of culture media used in CCP24 was 1 mL, 25 mL (CT50 and TCF) and 40 mL (EF). All vessels were placed in BD GasPak™ EZ container, charged with CampyGen 3.5 L (Oxoid) to produce microaerobic conditions and then incubated at 37 °C for 48 h. The growth rate of *C. hepaticus* HV10 was determined by the plate count method on HBA plates.

### Effect of pH and initial inoculum on *C. hepaticus* growth

The pH of Brucella broth was adjusted from 6.0 to 8.0 in increments of 0.5 units using 1 M NaOH or 1 M HCl. *C. hepaticus* was cultured in CCP24 plates to examine growth at different pH levels. Each well of CCP24 was inoculated with 10^6^ CFU/mL of *C. hepaticus* HV10^T^. Plates were incubated under microaerobic conditions at 37 °C. Growth was enumerated after 48 h of incubation by plating serial dilution on HBA. The experiment was performed in triplicate.

The effect of initial inoculum size on the growth kinetics and final culture yields was analysed using seven initial inoculum levels (10^1^ to 10^7^ CFU/ml) of *C. hepaticus* HV10^T^ in a CCP24 plate. The plates were incubated at 37 °C under microaerobic conditions. The growth of *C. hepaticus* HV10^T^ was examined after 24, 48, 60, 72, and 96 h of incubation using the plate count method on HBA.

### Growth of *C. hepaticus* in static and shaking conditions

*C. hepaticus* HV10^T^ was suspended into Brucella broth to achieve acell density of 10^5^ CFU/ml and then 1 mL of bacterial suspension was incubated into each well of CCP24 at static condition. For shaking conditions, the anaerobic jars were shaken in a shaking incubator with a speed of 100 rpm. After 48 h viable *C. hepaticus* cells were enumerated on HBA plates. The shaking conditions were only used for this test; static growth conditions were used at all other times.

### Effects of sodium pyruvate and amino acids on the growth of *C. hepaticus*

Based on the results of in silico pathway analysis of *C. hepaticus* and results from Alazzam et al.^14^, the following supplements were added to Brucella broth to examine the growth of this bacterium: amino acids (l-cysteine, l-lysine, l-methionine, and l-leucine, l-glutamine, l-valine, l-histidine); carbon source (sodium pyruvate) (Sigma). Each compound was completely dissolved in Milli-Q water at a concentration recommended in MCLMAN, a new minimal medium for *C. jejuni*^14^. The chemical solution was passed through a 0.22 µm syringe filter and stored at − 20 °C if not used immediately. On the day of the experiment, each substrate was added into a bacterial culture and then 1 mL transferred to each well of CCP plates. The plates were incubated under microaerobic conditions at 37 °C for 48 h.

### Effect of temperature on growth of *C. hepaticus*

Growth of *C. hepaticus* was tested at 37 °C and 42 °C in Brucella broth. Briefly, *C. hepaticus* cells were harvested from an HBA plate and suspended into Brucella broth to obtain an OD of 0.01–0.03. The bacterial suspension was supplemented with sodium pyruvate (10 mM), l-cysteine (0.4 mM), and l-glutamine (4 mM) (based on the results from the experiments described above). Bacterial culture (1 mL) was placed in CCP plates and incubated under microaerobic conditions for 48 h at 37 °C and 42 °C.

### Animal trial

A *C. hepaticus* chicken challenge trial was carried out to compare the virulence between *C. hepaticus* HV10^T^ cultures grown from modified Brucella broth developed in this study (liquid method) and the HBA plates (plate method). The plate method was described by Van et al.^10^ in which *C. hepaticus* HV10^T^ stored at − 80 °C in glycerol stocks was first streaked on to HBA plates, then further subcultured to HBA plates and cells were harvested and resuspended in Brucella broth to obtain 1 × 10^9^ CFU/mL. For the liquid method, *C. hepaticus* was grown in Brucella broth supplemented with l-cysteine (0.4 mM), and l-glutamine (4 mM) and sodium pyruvate (10 mM) in TCF75 at 37 °C for 48 h in microaerophilic conditions and used directly for the challenge. The animal experimentation was approved by the Wildlife and Small Institutions Animal Ethics Committee of the Victorian Department of Economic Development, Jobs, Transport and Resources (approval number 14.16). A total of 36 Hy-Line brown laying hens were used in the experiment. Only healthy birds laying eggs regularly were included in the study. Chickens were housed in groups of 4 birds per pen, with 3 pens per group: a total of 12 birds in each group (n = 12). Birds were randomly allocated to groups and cages by stratified rank order based on weight. Unchallenged control birds were orally inoculated with 1 ml of Brucella broth only whereas 1 ml of Brucella broth containing 1 × 10^9^ CFU of *C. hepaticus* HV10^T^ was orally administered to the birds for the plate and broth methods groups. Birds were sacrificed after 5 days and SLD lesions on the surface of the liver were counted to measure the severity of the induced disease. The experienced and trained scorers were blinded to the treatment groups. Scores were based on a logarithmic scale from 0 to 4, where 0 = no visible lesions, 1 = 1–9 lesions, 2 = 10–99 lesions, 3 = 100–999 lesions and 4 = more than 1000 lesions.

### Statistical analysis

All experiments to study the growth of *C. hepaticus* HV10 in different conditions and supplements were repeated a minimum of three times with biological replicates. *Campylobacter* cell counts in all tests were converted to log_10_ CFU/mL. Statistical comparison of all parameters was performed by t-test, one-way ANOVA using Graphpad Prism version 8 for Windows, GraphPad Software (San Diego California USA, www.graphpad.com). The significance level was set at 5% (p ≤ 0.05). Sample size calculation for the SLD animal trial was performed using the online calculator at https://clincalc.com/stats/samplesize.aspx.

### Declarations

All the methods were carried out in accordance with relevant guidelines and regulations and the animal trial is reported in accordance with the ARRIVE Essential 10 guidelines.
